# Investigating the Effect and Potential Mechanism of Rhamnetin 3-*O*-α-Rhamnoside on Acute Liver Injury In Vivo and In Vitro

**DOI:** 10.3390/ph18010116

**Published:** 2025-01-17

**Authors:** Dandan Deng, Borong Zhao, Hong Yang, Songsong Wang, Ziying Geng, Jiangtao Zhou, Guane Yang, Liwen Han

**Affiliations:** 1 School of Pharmaceutical Sciences, Shanxi Medical University, No. 56 South Xinjian Road, Taiyuan 030001, China; dengdandan@sxmu.edu.cn (D.D.); zhaoborong@sxmu.edu.cn (B.Z.); yanghong67@sxmu.edu.cn (H.Y.); gengziying@sxmu.edu.cn (Z.G.); zjt881206@sxmu.edu.cn (J.Z.); 2 School of Pharmaceutical Sciences & Institute of Materia Medica, Shandong First Medical University & Shandong Academy of Medical Science, No. 6699 Qingdao Road, Jinan 250117, China; wangsongsong@sdfmu.edu.cn

**Keywords:** rhamnetin 3-*O*-α-rhamnoside, acute liver injury, zebrafish, IKKβ/NF-κB signaling pathway, nuclear translocation, thioacetamide, oxidative stress

## Abstract

**Background/Objectives**: Rhamnetin 3-*O*-α-rhamnoside (ARR) is a major flavonoid of the herb *Loranthus tanakae* Franch. & Sav., which has been used for treating liver diseases in China. However, the protective effect of ARR on the liver has not been reported. **Methods**: Zebrafish larvae were used as a visual animal model, and liver injury was induced by thioacetamide (TAA) for an acute liver injury (ALI) model. The hepatoprotective activity of ARR was evaluated by assessing liver morphology, liver function indices, oxidative stress, and the mRNA expression levels of inflammation-related genes in the zebrafish model. Additionally, the ROS level, inflammatory factors, and protein expression related to the IKKβ/NF-κB signaling pathway were measured to investigate a potential mechanism of ARR in HepG2 cells. **Results**: ARR ameliorated TAA-induced growth retardation, reduced liver injury phenotypes, and decreased oxidative stress in the zebrafish. ARR was also able to lower ROS levels in HepG2 cells, effectively inhibit the overactivation of the IKKβ/NF-κB signaling pathway in pathological conditions, inhibit NF-κB p65 translocation from the cytoplasm to the nucleus, and reduce the release of intracellular inflammatory factors. **Conclusions**: ARR showed significant protective activity against TAA-induced liver injury in in vivo and in vitro models, and its potential mechanism was closely related to the IKKβ/NF-κB signaling pathway.

## 1. Introduction

Acute liver injury (ALI) results from hepatocellular damage due to various causes [1,2,3], which can trigger explosive and fatal liver failure in severe cases [4,5]. In clinical practice, hepatocyte membrane repair and protective agents, anti-inflammatory drugs, and antioxidants are commonly administered for pharmacotherapy. In severe cases, surgical intervention, such as liver transplantation, may be necessary. However, due to the complex etiology of ALI, there remains an urgent need for drugs with high specificity and safety in clinical treatment. Traditional Chinese medicine (TCM) comprises practical medicine based on clinical application, and the discovery of novel active compounds against ALI from TCM and related medicinal plants has garnered increasing attention from researchers. Some studies have found that active ingredients such as silymarin [6] and geraniol [7] found from TCM show great potential for the treatment of liver injury. Therefore, the search for some active ingredients from Chinese herbs will provide new ideas for the prevention and treatment of ALI.

*Loranthus tanakae* Franch. & Sav. belongs to the *Loranthaceae* family and is distributed in China, Korea, Japan, and other Asian countries [8]. Its stems and branches are commonly used in medicine as a substitute for the traditional Chinese medicine *Taxillus chinensis* [9], which has been documented for its effects on tonifying the liver and kidneys and dispelling wind-dampness in Ancient Chinese medical books [10,11]. Our preliminary research suggested that rhamnetin 3-*O*-α-rhamnoside (ARR) is the predominant flavonoid in *L. tanakae*, accounting for approximately 70% of its total flavonoid content [12]. However, whether ARR is the critical pharmacological agent responsible for the hepatoprotective effect of *L. tanakae* remains unclear.

Imbalance in the body’s redox system is a hallmark of ALI pathogenesis [13]. Oxidative stress reflects an imbalance between the production of reactive oxygen species (ROS) and the scavenging capacity of the antioxidant system [14]. ROS at high concentrations cause oxidative modification of cellular macromolecules (lipids, etc.) and lead to the accumulation of damaged macromolecules, which can cause liver injury and inflammatory responses [15]. Furthermore, increasing evidence suggests that reducing inflammation plays a crucial role in the therapeutic mechanism for liver injury [16,17]. The IKKβ/NF-κB signaling pathway is essential for processes such as cellular inflammation and immune response [18,19], and it regulates the onset of various diseases, including cancer [20], rheumatoid arthritis [21], and atherosclerosis [22]. Notably, the ethanol extract of *L. tanakae* effectively reduced the release of inflammatory cytokines in the lung tissues of COPD mice [23]. Therefore, we hypothesize that ARR may exert its pharmacological activity through its anti-inflammatory effects.

Thioacetamide (TAA) is a common hepatotoxic drug. In this study, we used TAA-induced liver injury in zebrafish to rapidly assess the hepatoprotective activity of ARR and investigated its potential to intervene in ALI by preserving the balance of the antioxidant system and modulating the inflammatory response, in conjunction with the HepG2 cell model. We anticipate that these findings will provide novel scientific evidence for the discovery of active substances that can be used to treat ALI.

## 2. Results

### 2.1. Effect of TAA on Liver Morphology of Zebrafish Larvae

**Figure 1:**
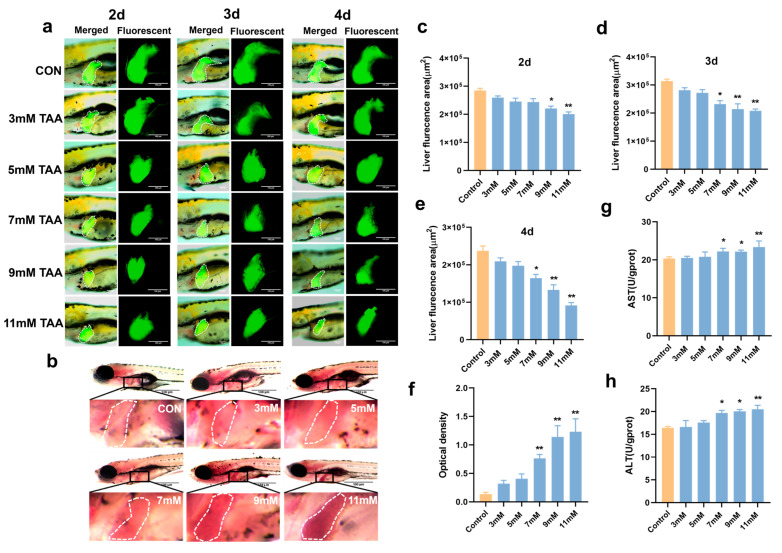
Effects of TAA on the liver of zebrafish larvae. (**a**) Effect of TAA on liver fluorescence in Tg (*−1.7apoa2:GFP*) zebrafish larvae. Scale bar = 100 μm. (**b**) Oil Red O staining of wild-type AB zebrafish liver (*n* = 10). Scale bar = 100 μm. (**c**–**e**) Changes in fluorescent liver area of larvae at 2, 3, and 4 days following TAA treatment. (**f**) Statistics of lipids in the wild-type zebrafish liver. (**g**) The activity of AST in the wild-type zebrafish. (**h**) The activity of ALT in the wild-type zebrafish. * *p* < 0.05, ** *p* < 0.01 vs. control (mean ± SEM).

The Tg (−*1.7apoa2:GFP*) zebrafish is a transgenic line expressing green-fluorescent protein reporter genes driven by the 14 kDa apolipoprotein (Apo-14) promoter sequence. The continuous expression of green-fluorescent protein driven by the Apo-14 promoter in the hepatocytes, hepatic progenitor cells, and liver cells of both liver primordia and zebrafish allows for the detailed observation and documentation of liver organogenesis [24]. A transgenic Tg (−*1.7apoa2:GFP*) zebrafish with a green-fluorescently labeled liver was used to establish a TAA-induced ALI model. Following TAA treatment, zebrafish larvae exhibited liver damage to a certain extent (Figure 1a). The fluorescent area of the liver in the TAA-treated group decreased compared with the control group. This reduction in fluorescence was dose-dependent and became more pronounced with higher TAA concentrations and longer exposure times (Figure 1a,c–e), indicating that TAA caused liver damage in the larvae. By 6 days post fertilization (dpf), the yolk sac was insufficient to provide nutrients for the larvae, necessitating exogenous food for proper nutrition. In order to stop food from interfering with the experiment, we selected 7 mM TAA to treat larvae at 72 h post-fertilization (hpf) for 3 days. To further verify the validity of this modeling condition, a liver enzyme activity assay and Oil Red O staining assay were utilized for relevant verification.

After 3 days of treatment with 7 mM TAA, AST and ALT levels were measured in each group (Figure 1g,h). Compared with the control group, the AST and ALT levels showed a significant increase, starting at 7 mM (*p* < 0.05). The Oil Red O staining results indicated a significant increase in lipid accumulation in the zebrafish liver at a concentration of 7 mM and above (*p* < 0.05) (Figure 1b,f). These findings indicated that the specified conditions successfully established a TAA-induced zebrafish liver injury model.

### 2.2. ARR Inhibits TAA-Induced Developmental Toxicity in Zebrafish

**Figure 2:**
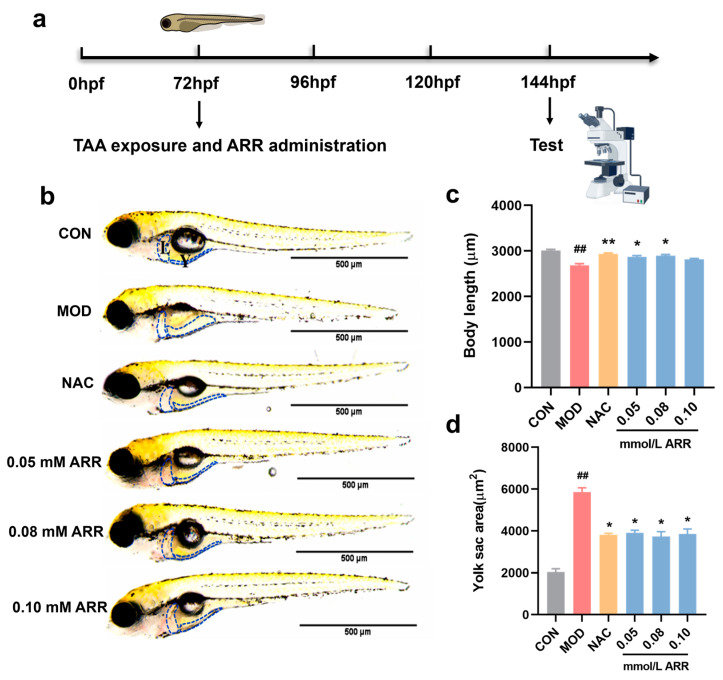
Effects of ARR on TAA-induced zebrafish larvae developmental toxicity. (**a**) The 72 hpf wild-type AB zebrafish larvae were handled for 3 days (*n* = 15). (**b**) Effects of ARR on the body length and yolk sac of zebrafish induced by TAA. Scale bar = 500 μm. (**c**) The change in body length. (**d**) The change in the absorption area of the yolk sac. The blue box indicates the location of the liver and yolk sac. L: liver; Y: yolk sac. * *p* < 0.05, ** *p* < 0.01 vs. control; ## *p* < 0.01 vs. model (mean ± SEM).

The phenomenon of yolk sac uptake is used as a test when evaluating the hepato-protective effects of drugs on zebrafish. In this study, the protective effect of ARR was assessed by measuring body length changes in zebrafish in relation to the phenomenon of yolk sac resorption. Zebrafish larvae were exposed to a 7 mM TAA solution at 72 hpf and simultaneously administered ARR at concentrations of 0.05, 0.08, and 0.10 mM. Above 0.20 mM, ARR caused deformities and some mortality in the zebrafish (Figure S1). After 3 days of TAA treatment, the larvae exhibited an obviously smaller body length compared with the control group, and the yolk sac absorption area was significantly larger. *N*-acetylcysteine (NAC) is a well-known antioxidant and can be used clinically as a therapeutic agent for liver injury [25]. In this experiment, NAC was used as a positive control. Compared with the TAA group, the growth and development of zebrafish in the positive control NAC group were significantly improved (*p* < 0.05). With the addition of ARR, the growth and development of the larvae improved significantly (*p* < 0.05). The body length of the larvae reached the same level as that of the control group, and the delay in yolk sac absorption was considerably reduced (Figure 2a–c). These findings suggest that ARR can attenuate TAA-induced developmental toxicity in larvae.

### 2.3. ARR Alleviates TAA-Induced Liver Injury in Zebrafish

**Figure 3:**
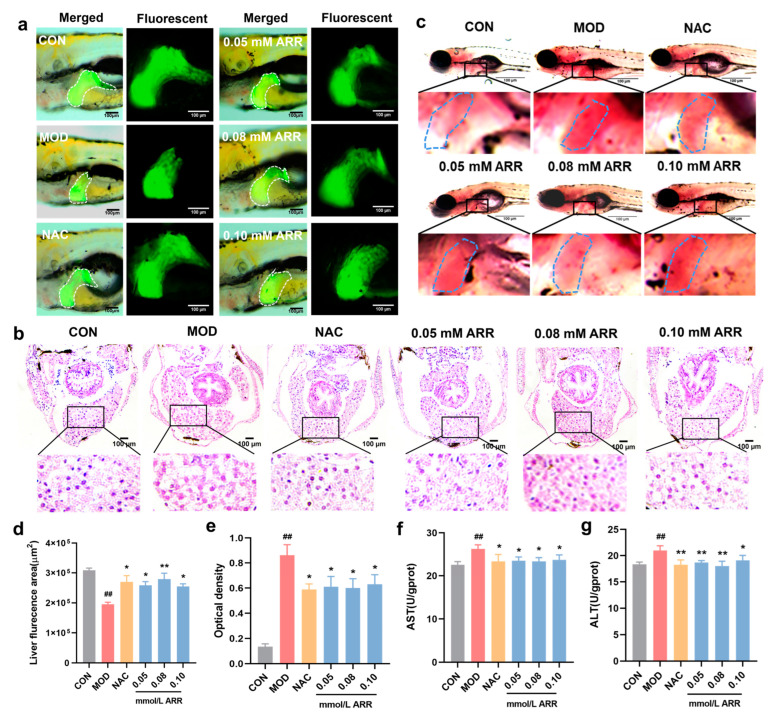
Effects of ARR on TAA-induced liver injury. (**a**) Effect of ARR on the fluorescence area of the liver of Tg (*−1.7apoa2:GFP*) zebrafish larvae exposed to TAA. (**b**) HE staining of wild-type AB zebrafish larvae. (**c**) Oil Red O staining of wild-type AB zebrafish larvae (*n* = 10). (**d**) Liver fluorescence area statistics for each group. (**e**) Statistics of lipids in the liver. (**f**) The activity of AST. (**g**) The activity of ALT. Scale bar = 100 μm. * *p* < 0.05, ** *p* < 0.01 vs. control; ## *p* < 0.01 vs. model (mean ± SEM).

In order to assess whether ARR could mitigate TAA-induced hepatotoxicity in zebrafish larvae, we examined liver morphology, histopathological sections, liver function indices, and lipid droplet status in each group. Compared with the control group, the liver area of the TAA-treated zebrafish larvae was significantly reduced. However, with the addition of ARR and NAC, the liver area increased (*p* < 0.05). Further, a trend of initial increase followed by a decrease in the liver area was observed at ARR concentrations of 0.05–0.10 mM (Figure 3a,d). H&E staining revealed that hepatocytes in the TAA group were tightly packed with larger intercellular gaps and extensive vacuolization compared with the control group. In contrast, the ARR and NAC groups exhibited densely arranged hepatocytes with a more compact cytoplasm, suggesting an ameliorative effect on TAA-induced liver tissue damage (Figure 3b). Oil Red O staining was employed to statistically analyze the mean optical density of liver regions in each group. The liver tissue in the TAA group showed a higher accumulation of lipid droplets (Figure 3c), with a significantly higher mean optical density. The ARR group, compared with the TAA group, had a significantly lower average optical density in the liver region (*p* < 0.05), indicating reduced lipid accumulation.

ALT and AST serve as crucial markers of hepatocyte damage. Elevated levels of these enzymes indicate hepatocyte pathology. Enzymatic activity assays demonstrated that the AST and ALT levels in zebrafish larvae were significantly upregulated following TAA exposure, indicating liver tissue injury (Figure 3f,g). Subsequent ARR and NAC administration led to a significant reduction in the activities of both enzymes (*p* < 0.05).

### 2.4. ARR Relieves TAA-Induced Oxidative Stress Levels in Zebrafish Larvae

**Figure 4:**
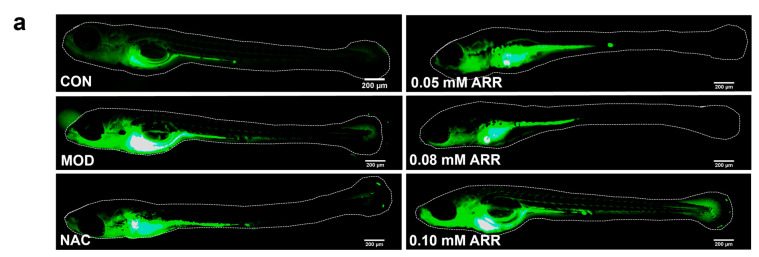

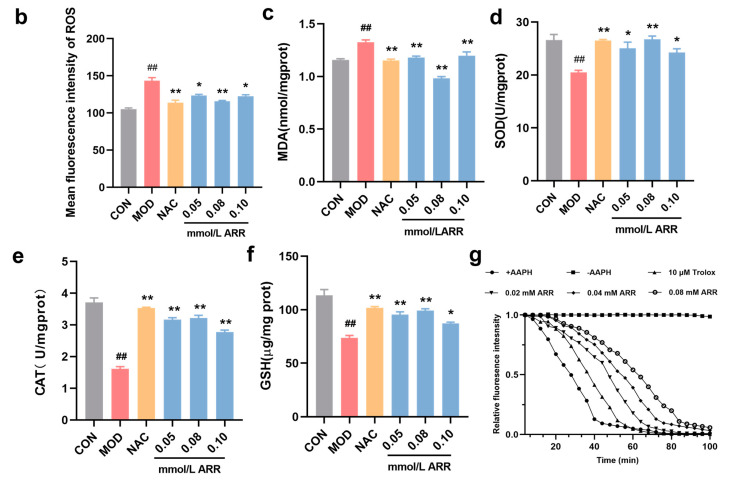
Effects of ARR on oxidative stress induced by TAA in wild-type AB zebrafish. (**a**) Effect of ARR on the activity of ROS (*n* = 15). (**b**) Mean optical density of ROS. (**c**) Content of MDA. (**d**) Activity of SOD. (**e**) Activity of CAT. (**f**) Content of GSH. (**g**) Fluorescence-quenching kinetics curve of ARR in ORAC experiment. Scale bar = 200 μm. * *p* < 0.05, ** *p* < 0.01 vs. control; ## *p* < 0.01 vs. model (mean ± SEM).

Changes in oxidative stress levels serve as critical biological markers of homeostasis disruption in organisms. Exposure to external stimuli disrupts the endogenous ROS balance, leading to an overproduction of free radicals that inflict severe cellular damage and induce apoptosis [26]. To detect ROS, we used 10 μM DCFH-DA staining and observed a significant increase in ROS fluorescence levels in the TAA group compared with the control. After TAA treatment, NAC was added, which significantly reduced the green-fluorescence intensity of ROS. Similarly, following the ARR intervention, the ROS fluorescence levels decreased significantly (*p* < 0.05) (Figure 4a,b). Oxidative stress index measurements revealed that 7 mM TAA significantly reduced the activities of SOD, CAT, and GSH and increased the MDA content in the larvae. During the TAA treatment, after adding NAC, the contents of SOD, CAT, and GSH in the zebrafish larvae increased significantly, and the content of MDA decreased (*p* < 0.05). This may be due to the conversion of part of the NAC into glutathione, increasing the antioxidant content in the zebrafish and improving their ability to remove ROS. And NAC can also provide mercapto groups for liver enzymes [25], which may play a key role in promoting the enzyme activity of the liver and maintaining the metabolism of the liver, thus alleviating TAA-induced oxidative stress. Following ARR treatment, normal levels of SOD, CAT, and GSH were recovered and a significant reduction in MDA content was observed (*p* < 0.05) (Figure 4c–f). These results demonstrate that ARR can effectively modulate ROS, MDA, SOD, CAT, and GSH levels, enhance antioxidant capacity, and mitigate the oxidative stress response induced by TAA in zebrafish larvae, thereby ameliorating liver injury.

Oxygen Radical Absorbance Capacity (ORAC) is a direct measure of oxygen radical uptake capacity. The ORAC assay experiment on ARR provided insight into the potential of ARR to scavenge free radicals in vitro. In the experiment, the standard curve for Trolox was *Y* = 0.2631*X* + 4.7995 (*R*^2^ = 0.9947). The kinetic curve of the fluorescence-quenching kinetics of ARR is shown in Figure 4g. It can be seen from the figure that with the increase in concentration, the effect of ARR on delaying fluorescence-quenching increases. The ORAC value of 0.04 mM ARR measured according to the standard curve was 746.31 μmol TE/g.

### 2.5. ARR Reduces Neutrophil Migration and Inhibits the Expression of Genes Related to NF-κB Signaling Pathway

**Figure 5:**
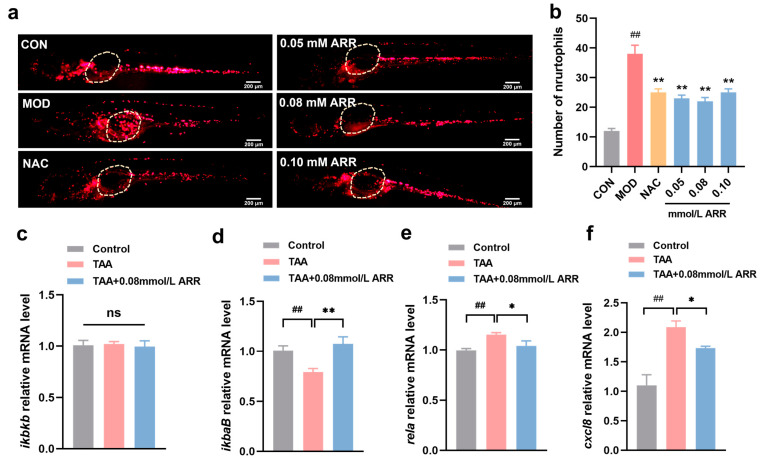
Effects of ARR on TAA-induced inflammation. (**a**) Effect of ARR on neutrophil migration in Tg (*lyz:DsRED2*) transgenic zebrafish larvae (*n* = 15). White color indicates the position of the yolk sac and swim bladder. (**b**) Statistics of the number of migrating neutrophils. (**c**) Expression level of *ikbkb*. (**d**) Expression level of *ikbaB*. (**e**) Expression level of *rela*. (**f**) Expression level of *cxcl8*. Scale bar = 200 μm. *n* = 3. * *p* < 0.05, ** *p* < 0.01 vs. control; ## *p* < 0.01 vs. model; ns: *p* > 0.05 (mean ± SEM).

Tg (*lyz:DsRED2*) transgenic zebrafish, which exhibit neutrophil-specific fluorescence, were used to observe neutrophil migration, indicating the location of inflammation [27]. In contrast to previous inflammatory responses, significant migration of neutrophils to the swim bladder and yolk sac of the larvae was observed following TAA exposure, suggesting inflammation at these sites. When ARR and NAC were administered, the number of neutrophil migrations significantly decreased (Figure 5a,b).

To determine the effect of ARR on the expression of inflammation-related genes in the TAA-induced liver injury model, we assessed the mRNA expression levels of genes associated with the NF-κB signaling pathway. Compared with the control group, the mRNA expression levels of *rela* (NF-κB p65) and *cxcl8* (IL8) were upregulated, whereas the expression level of *ikbaB* (IκBα) was significantly downregulated in the TAA group (Figure 5d–f). The expression level of *ikbkb* (IKKβ) tended to increase; however, the difference was not significant (Figure 5c). In the 0.08 mM ARR-treated group, the *rela* and *cxcl8* mRNA expression levels were significantly lower than in the TAA group, whereas the decrease in *ikbkb* mRNA expression was not notable (*p* < 0.05). Additionally, ARR upregulated the mRNA expression level of *ikbaB*. These results suggest that ARR may inhibit the activation of the NF-κB signaling pathway, thus attenuating the inflammatory response.

### 2.6. ARR Decreases TAA-Stimulated ROS Production and Inflammatory Factor Content in HepG2 Cells

**Figure 6:**
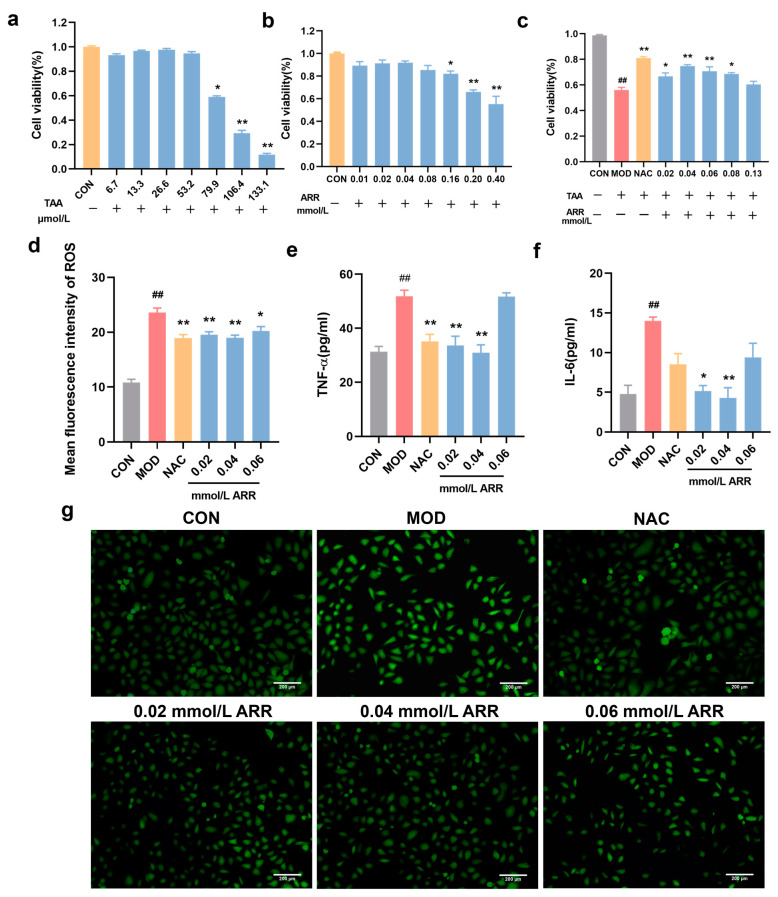
Effects of ARR on TAA-induced levels of ROS and inflammatory factors following TAA induction. (**a**) Effect of TAA on the viability of HepG2 cells. (**b**) Effect of ARR on the viability of HepG2 cells. (**c**) Effect of ARR on TAA-induced survival of HepG2 cells. (**d**) ROS levels. (**e**) Content of TNF-α. (**f**) Content of IL-6. (**g**) Effect of ARR on TAA-induced ROS levels in HepG2 cells. Scale bar = 200 μm. *n* = 3. * *p* < 0.05, ** *p* < 0.01 vs. control; ## *p* < 0.01 vs. model (mean ± SEM).

HepG2 cells come from a human-derived hepatocellular carcinoma cell line with a simple culture and stable genetic background, which is frequently used in liver injury or fatty liver models. The cell viability at ARR concentrations of 0.01, 0.02, 0.04, and 0.08 mM was 89.37%, 91.26%, 92.39%, and 85.59%, respectively, showing no statistically significant difference compared with the control group (Figure 6b). However, at ARR concentrations of 0.16, 0.20, and 0.40 mM, the cell viability decreased significantly (*p* < 0.05), indicating a dose-dependent effect (Figure 6c). Consequently, 0.02, 0.04, and 0.06 mM ARR were selected for subsequent experiments. Following TAA stimulation, HepG2 cell survival decreased to 56.13% of that of the blank control. After the addition of NAC, the cell viability of HepG2 was significantly improved. Then, treatment with 0.02, 0.04, and 0.06 mM of ARR significantly improved cell viability (*p* < 0.05).

HepG2 cells were stimulated with TAA, and intracellular ROS fluorescent levels were measured for each group to evaluate whether ARR exhibited a better antioxidant capacity at the cellular level. Compared with the control group, HepG2 cells in the TAA group exhibited an enhanced intensity of green fluorescence, indicating that TAA induced oxidative damage in the HepG2 cells (Figure 6d,g). In contrast, treatment with 0.02, 0.04, and 0.06 mM of ARR resulted in a dose-dependent decrease in ROS fluorescence intensity (*p* < 0.05), suggesting that ARR inhibits TAA-induced oxidative damage in HepG2 cells.

Additionally, we measured the levels of inflammatory factors. Following TAA induction, the TNF-α and IL-6 levels in the cell culture supernatants increased significantly. After adding 10 µM NAC, the content of TNF-α was significantly reduced, but the reduction level of IL-6 was not obvious. At ARR concentrations of 0.02 and 0.04 mM, the levels of TNF-α and IL-6 in the supernatant were significantly lower than those in the TAA group (*p* < 0.05), with a more pronounced reduction than that observed in the positive drug group. However, in the 0.06 mM ARR group, the TNF-α and IL-6 levels did not significantly differ from those in the TAA group (Figure 6e,f). These results confirmed that ARR could mitigate TAA-induced local inflammation in zebrafish. Notably, the upregulation of TNF-α and IL-6 following TAA stimulation suggests the possible activation of the NF-κB signaling pathway.

### 2.7. ARR Inhibits the Activation of the IKKβ/NF-κB Signaling Pathway

**Figure 7:**
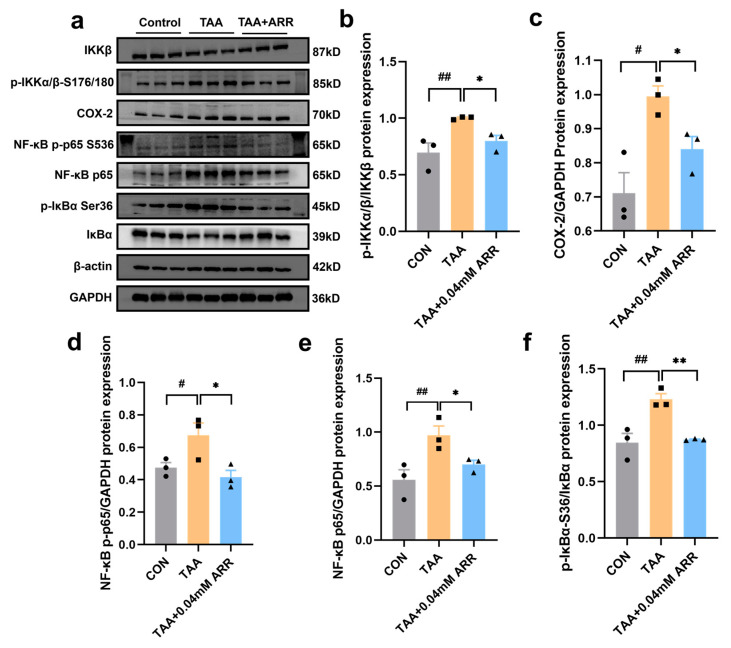
Effects of ARR on TAA-induced activation of IKKβ/NF-κB signaling pathway. (**a**) Expression levels of IKKβ, p-IKKα/β, COX-2, NF-κB p-p65, NF-κB p65, p-IκBα, IκBα, and GAPDH detected using Western blotting. (**b**) Expression level of p-IKKα/β/IKKβ. (**c**) Expression level of COX-2. (**d**) Expression level of NF-κB p-p65. (**e**) Expression level of NF-κB p65. (**f**) Expression level of p-IκBα/IκBα. *n* = 3. * *p* < 0.05, ** *p* < 0.01 vs. control; # *p* < 0.05, ## *p* < 0.01 vs. model (mean ± SEM).

NF-κB plays a critical role in inflammation. Therefore, we examined the expression of proteins related to the IKKβ/NF-κB signaling pathway in HepG2 cells to determine how ARR suppressed NF-κB activation using Western blotting. Notably, the expression levels of NF-κB p65, NF-κB p-p65, COX-2, p-IκBα, and p-IKKα/β proteins were significantly upregulated in the TAA group compared with the control group (*p* < 0.05). However, 0.04 mM ARR significantly suppressed the phosphorylation of NF-κB p65, IκBα, and IKKα/β in TAA-treated HepG2 cells (Figure 7a–e) and concurrently decreased the expression level of COX-2 (*p* < 0.05) (Figure 7a,c). The anti-inflammatory effect of ARR may be attributed to the inhibition of IKKβ/NF-κB signaling pathway activation.

### 2.8. ARR Prevents Nuclear Translocation of NF-κB p65

**Figure 8:**
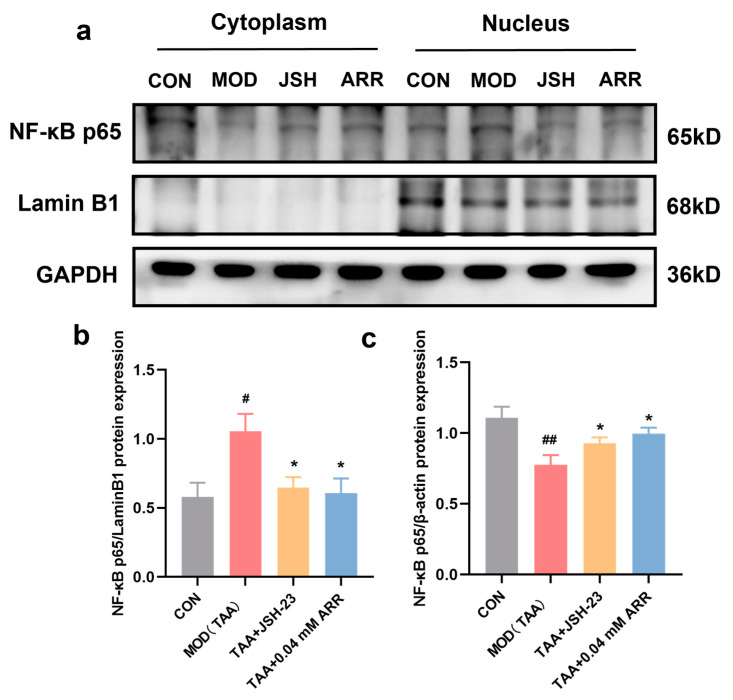
Effect of ARR on nuclear translocation of NF-κB p65. (**a**) Expression of the nuclear and cytoplasmic protein NF-κB p65. (**b**) Level of NF-κB p65 expression in the nucleus. (**c**) Level of NF-κB p65 expression in the cytoplasm. *n* = 3. * *p* < 0.05vs. control; # *p* < 0.05, ## *p* < 0.01 vs. model (mean ± SEM).

To assess whether ARR could inhibit the nuclear translocation of NF-κB p65 and thereby reduce the inflammatory response, we extracted cytoplasmic and nuclear proteins to measure NF-κB p65 expression. Notably, following the TAA stimulation of HepG2 cells, the nuclear content of the NF-κB p65 protein was significantly elevated compared with the control group (*p* < 0.05). JSH-23 is an NF-κB-specific chemical inhibitor that blocks the translocation of NF-κB p65 to the nucleus and was used in this experiment as a positive control. After the use of JSH-23, the expression of NF-κB p65 in the nucleus after TAA treatment was significantly reduced, and its expression in the cytoplasm was increased (*p* < 0.05). Further, compared to the TAA group, the nuclear content of the NF-κB p65 protein significantly decreased after treatment with 0.04 mM ARR (Figure 8a,b). Conversely, the cytoplasmic NF-κB p65 protein levels were significantly reduced after TAA stimulation (*p* < 0.05) (Figure 8a,c), whereas the 0.04 mM ARR treatment significantly increased cytoplasmic NF-κB p65 protein expression (*p* < 0.05). Under normal conditions, the NF-κB p65 protein predominantly resides in the cytoplasm. TAA stimulation prompted a gradual relocation of NF-κB p65 from the cytoplasm to the nucleus. However, intervention with ARR prevented the nuclear translocation of NF-κB p65, suggesting a potential mechanism for mitigating cellular inflammation.

## 3. Discussion

Genetic information about the liver and immune system in zebrafish is highly similar to that for humans [28,29]. Moreover, zebrafish larvae are semi-transparent, and their liver cells contain special fluorescent markers [30], enabling the direct observation of drug effects. Consequently, they are frequently used to simulate liver disease models [31]. TAA can generate toxic metabolites that accumulate in the liver through the action of the CYP450 2E1 system [32,33]. This system mediates the onset of oxidative stress [34], leading to hepatocyte injury and necrosis [35]. Zebrafish serve as a holistic animal model, providing a complete internal environment for studying liver function. Additionally, the rapid growth rate of zebrafish allows for the completion of experiments in a very short time. In this study, we constructed an acute liver injury model using zebrafish, which offered a convenient and rapid animal model to investigate the hepatoprotective activity of ARR.

ARR exhibited significant hepatoprotective effects in the zebrafish model. Isolated from *L. tanakae*, ARR is a flavonoid compound characterized by low biotoxicity and potent anti-inflammatory pharmacological activity [36]. Its efficacy in combating and treating liver injury has not been previously reported. This study demonstrated that ARR enhanced the growth and development of larvae exposed to TAA, repaired liver damage, and reduced the accumulation of lipid droplets in hepatic tissue, thereby exerting a protective effect on the liver. Our research unveils the considerable potential of ARR in the prevention and treatment of liver damage.

ARR alleviated oxidative stress levels and mitigated liver damage. Oxidative stress is a complex phenomenon leading to cytotoxicity [15]. A study demonstrated that low concentrations of long-term TAA induction reduced the levels of G6PDH and SOD, subsequently promoting the expression of Bax and P53, which caused apoptosis in zebrafish hepatocytes, resulting in liver damage [37]. In the present study, the zebrafish larvae produced an excessive amount of ROS, and SOD, CAT, and GSH activities were significantly reduced, whereas the content of MDA increased significantly after TAA stimulation. Consequently, the reduction in key substances in the antioxidant system increased lipid peroxidation and decreased the antioxidant capacity of the body, ultimately exacerbating liver damage. In the ORAC assay, the results showed that the effect of delayed fluorescence-quenching increased with increasing ARR concentration, suggesting that ARR showed good oxygen radical uptake capacity in vitro. ARR revealed an apparent antioxidant capacity in both the in vivo and ex vivo experiments, which may attenuate TAA-induced hepatotoxicity by regulating the level of oxidative stress.

**Figure 9:**
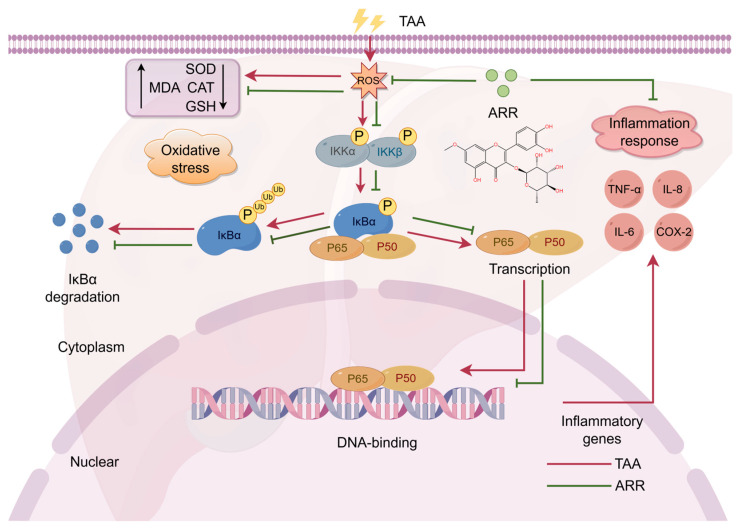
Mechanism of TAA-induced activation of IKKβ/NF-κB signaling pathway inhibition by ARR.

ARR may exert its hepatoprotective effect by inhibiting the activation of the IKKβ/NF-κB signaling pathway. Notably, TAA triggers oxidative stress responses in the body [38] and stimulates the release of pro-inflammatory cytokines such as TNF-α and IL-6, thereby activating the classical NF-κB signaling pathway [39]. In the IKKβ/NF-κB signaling pathway, the *rela*, *ikbaB*, *cxcl8,* and *ikbkb* genes regulate the NF-κB, IκBα, IL-8, and IKKβ proteins, respectively. NF-κB, a crucial intracellular nuclear transcription factor, is associated with various human diseases, including liver disease, inflammation, and autoimmune disorders [40]. IKKβ, the catalytic subunit of the IKK complex, is critical in the classical pathway of NF-κB activation. The knockdown of IKKβ inhibited TNF-induced NF-κB activation in the primary hepatocytes of mice [41]. Therefore, suppressing the IKKβ/NF-κB activation state may be a potential therapeutic target. A study reported that sitagliptin ameliorated TAA-induced liver injury in mice by reducing TLR4 and NLRP3 levels and negatively regulating the NF-κB signaling pathway [42]. ARR effectively prevented NF-κB p65 translocation activity in LPS-induced RAW264.7 cells and reduced the release of inflammatory mediators [36]. However, the specific effect of ARR on the pathological state of the IKKβ/NF-κB pathway remains unclear. In the present study, we found that the mRNA expression levels of *rela* and *cxcl8* were significantly upregulated, whereas the mRNA expression level of *ikbaB* was downregulated in the TAA group compared with normal zebrafish, indicating NF-κB pathway activation. Notably, similar results were observed in the HepG2 cell experiments. TAA activated the classical IKKβ/NF-κB signaling transduction pathway, with increased phosphorylation levels of IKKβ and IκBα, degradation of the IκBα zymosome, and nuclear translocation of NF-κB p65. Under the action of ARR, the activation of IKKβ/NF-κB signaling was blocked. And ARR shows the same effect as the inhibitor JSH-23. The translocation of NF-κB p65 to the nucleus was reduced, decreasing the release of inflammatory mediators such as TNF-α and COX-2 and simultaneously suppressing the inflammatory response. Therefore, these findings further suggest that ARR could reduce TAA-induced hepatotoxicity and promote liver development at the molecular level (Figure 9). Unlike other cells, the HepG2 cells showed changes in total NF-κB p65 protein and consistent trends in phosphorylation levels following ARR administration. These findings were also consistent with those of Chen et al. [43].

In summary, this study provides valuable experimental evidence for future pharmacological investigations of ARR. The results of this study will provide valuable experimental evidence for the study of the hepatoprotective pharmacological activity of ARR, suggesting that ARR can be used as a bio-functional ingredient in the future for the development of hepatoprotective nutraceuticals and pharmaceutical products. In this study, the hepatoprotective mechanism of ARR was not fully explored, and it is necessary to explore the molecular mechanism of ARR in combination with rats or other animal models from multiple perspectives and in depth. In addition, no significant quantitative relationship was shown between the doses of ARR in this study, which suggests that the effect of different quantities of ARR should be explored in depth in subsequent studies.

## 4. Materials and Methods

### 4.1. Chemical Reagents

Rhamnetin 3-*O*-α-rhamnoside, purity ≥ 95% (Figure S2), was extracted and isolated from *L. tanakae* in our laboratory according to a previous method [44,45]. Thioacetamide was purchased from Sigma-Aldrich (St. Louis, MO, USA). Detection kits for alanine aminotransferase (ALT), aspartate amino-transferase (AST), superoxide dismutase (SOD), catalase (CAT), and malondialdehyde (MDA) were obtained from Nanjing Jiancheng Bioengineering Institute (Nanjing, China). An ORAC kit was purchased from Congyi Technology Co., Ltd. (Shanghai, China). The kits for reduced glutathione (GSH) and reactive oxygen species were bought from Solarbio Science and Technology Co., Ltd. (Beijing, China). SparkZol Reagent (AC0101), SPARKscript II RT Plus Kit (AG0304), and 2×SYBR Green Qpcr Mix (AH0104) were purchased from Sparkjade Biotechnology Co., Ltd. (Shandong, China). The antibodies for NF-κB p65 (db11612), phospho-NF-κB p65 (db7996), IκBα (db11682), COX-2 (db14667), and IKKβ (db12051) were obtained from Diagbio Technology Co., Ltd. (Hangzhou, China). GAPDH (A19056), β-actin (AC026), Lamin B1 (A1910), phospho-IκBα (AP1220), and HRP-conjugated goat anti-rabbit IgG (AS014) antibodies were purchased from ABclonal (Wuhan, China). Phospho-IKKα/β-S176/180 antibody (2697T) was bought from Cell Signaling Technology (Danvers, MA, USA). JSH-23 was obtained fromMedChem Express (Monmouth Junction, NJ, USA). All other chemicals were of analytical grade.

### 4.2. Zebrafish Assay

#### 4.2.1. Modeling of Zebrafish Liver Injury

Wild-type AB zebrafish (*Danio rerio*) and Tg (−*-1.7apoa2:GFP*) transgenic lines, obtained from the Zebrafish Center of Shandong First Medical University, China, were used in this study. Adult zebrafish were maintained in standard conditions with 14/10 h light/dark cycles at 28 ± 0.5 °C and were fed twice daily with hatched brine shrimp. When the experiment started, male and female zebrafish were placed in a breeding tank separated by a spacer at a ratio of 1:1 or 1:2. The following morning, embryos were collected and washed with culture water within 1 h of spawning. All healthy fertilized eggs were incubated in a light-exposed incubator at 28 °C. At 72 hpf, normal larvae were randomly selected, assigned to the control group (including 1‰ DMSO) or one of the TAA groups (3, 5, 7, 9, and 11 mM), housed in 12-well plates (*n* = 30 per group) and, respectively, treated for 2, 3, or 4 days. At the end of the experiment, zebrafish larvae were anesthetized with Tricaine. The survival rate was recorded, and the fluorescent area in the ipsilateral liver of the larvae was imaged daily after exposure using an IX83 fluorescent microscope (Olympus, Tokyo, Japan). Meanwhile, the larvae were collected for overall Oil Red O staining with the liver enzyme activity assay. The results were analyzed with ImageJ.JS software with Java 8 (NIH, Bethesda, MD, USA).

#### 4.2.2. Determination of the ORAC of ARR

Following the kit’s instructions, the standard Trolox curve was drawn, with AUC as the ordinate. Fluorescence assay conditions: the excitation wavelength was 485 nm, the emission wavelength was 520 nm, the number of cycles was 25, and each cycle lasted 4 min. Then, the ORAC values at 0.02, 0.04, and 0.08 mM ARR were measured sequentially by a Microplate Multifunction Analyzer (Thermo Fisher Scientific, Waltham, MO, USA).

#### 4.2.3. ARR Treatment

ARR solution (200 mM) was dissolved in DMSO and stored at 4 °C. Healthy larvae at 72 hpf were placed in Petri dishes and treated with either DMSO control, 7 mM TAA (model), TAA + 10 μM NAC (positive control), or TAA + ARR (0.05, 0.08, and 0.10 mM) administration for 3 days. After 3 days, the larvae were collected for a series of analyses, including measurement of the green-fluorescent liver area, body length and yolk area, H&E staining, and Oil Red O staining (Yuanye Bio-Technology Co., Ltd., Shanghai, China).

#### 4.2.4. ROS Measurement

ROS content can be determined using a DCFH-DA probe (Solarbio, Beijing, China). Fifteen AB zebrafish larvae from each group were incubated with 10 µM DCFH-DA for 20 min in the dark. Thereafter, the larvae were rinsed with culture water, anesthetized with Tricaine, and photographed.

#### 4.2.5. Neutrophil Migration Analysis

Tg (*lyz:DsRED2*) transgenic zebrafish were used in this experiment. Upon completion of the experiment, the number of neutrophils in the larvae that migrated to the yolk sac and swim bladder regions was counted using ImageJ.JS.

#### 4.2.6. Related Enzyme Activity Measurements

The supernatant was prepared for protein concentration measurement using a BCA kit (Beyotime Biotechnology, Shanghai, China). The activities or levels of AST, ALT, CAT, SOD, MDA, and GSH were determined following the manufacturer’s instructions. The protein concentration of the supernatant was then used for normalization.

#### 4.2.7. Quantitative Real-Time PCR (qPCR) Analysis

Total RNA was extracted from 70 zebrafish larvae and reverse-transcribed to generate cDNA using the SPARKscript II RT Plus Kit. QPCR amplification was performed using the 2× SYBR Green Qpcr Mix, and the Roche LightCycler RT-PCR machine (Roche, Basel, Switzerland) was used to determine the expression levels of inflammation-related genes (Tables S1–S3). β-actin served as the internal reference gene, and the results were calculated using the 2^−ΔΔCt^ method. Amplification and dissolution curves are presented in Figure S3, and all primer sequences are listed in Table 1.

### 4.3. Cell Assay

#### 4.3.1. Cell Viability Assay

HepG2 cells (provided by Procell, Wuhan, China, CL-0103), human hepatocellular carcinoma cells, were seeded into 96-well plates at a density of 5 × 10^3^/100 μL, with 6 replicate wells. After the cells had completely attached to the well surface, the supernatant was removed, and the culture medium was replaced. The control group was supplemented with DMEM medium (Procell, Wuhan, China) containing 0.1% DMSO, the model group with TAA (79.9 μM), the positive control with TAA + 10 μM NAC, and the administration group with TAA + ARR (0.02, 0.04, 0.06, 0.08 and 0.13 mM). They were incubated for 24 h. Subsequently, 10 µL of CCK-8 reagent (Solarbio, Beijing, China) was added to each well, and the cells were incubated for an additional 2 h at 37 °C and 5% CO_2_. Cell viability was assessed by measuring the absorbance at 450 nm using a SpectraMax microplate reader (Molecular Devices, Shanghai, China).

#### 4.3.2. Intracellular ROS Assay

HepG2 cells in the logarithmic growth phase were seeded in 12-well plates. After 24 h of drug administration, 10 μM of a DCFH-DA probe solution was added to each well for 30 min in the dark. Subsequently, the wells were washed three times with basal medium to remove the solution. The fluorescence of each group was examined using an inverted fluorescence microscope (Ningbo Sunny Instruments Co., Ltd., Yuyao, China), and the average fluorescence intensity was quantified with ImageJ.JS.

#### 4.3.3. Inflammatory Factor Assay

The culture supernatant from HepG2 cells was collected, and the levels of tumor necrosis factor α (TNF-α; ABclonal) and interleukin 6 (IL-6; ABclonal) were measured using an enzyme-linked immunosorbent assay (ELISA) following the manufacturer’s instructions.

#### 4.3.4. Western Blotting Analysis

HepG2 cells were seeded into a 10 cm Petri dish, and the culture medium was replaced with the following for 24 h: DMEM medium (0.1% DMSO), TAA (79.9 μM), and TAA + ARR (0.04 mM). The JSH group (TAA + 20 μM JSH-23) was used as a positive control in the nuclear translocation experiments. Total protein, cytoplasmic proteins, and nuclear proteins were extracted using the Nuclear and Cytoplasmic Protein Extraction Kit (Beyotime Biotechnology, Shanghai, China). Proteins were quantified, denatured, and separated using SDS-PAGE. Proteins were then transferred onto a PVDF membrane, which was then blocked with 5% BSA. The membrane was incubated overnight at 4 °C with primary antibodies against NF-κB p65 (1:2000), NF-κB p-p65 (1:2000), IκBα (1:5000), p-IκBα (1:750), IKKβ (1:2000), p-IKKα/β (1:1000), COX-2 (1:2000), Lamin B1 (1:1000), β-actin (1:15,000), and GAPDH (1:10,000). Subsequently, HRP-conjugated secondary antibody was used to hybridize with the protein bands. Detection was performed using the Fluorescent and Chemiluminescence Gel Imaging System (JiaPeng Science Technology Co., Ltd., Hangzhou, China), and the membranes were stripped using a stripping buffer (Cwbio, Taizhou, China).

### 4.4. Statistical Analysis

SPSS 26.0 and GraphPad Prism 8.0.1 software was applied for data analysis and graphing, respectively. Multiple sample means were analyzed using a one-way ANOVA followed by Duncan’s method. *p* < 0.05 was considered statistically significant, and indicators for each group were expressed as the mean ± SEM.

## 5. Conclusions

In this research, we discovered the protective activity of ARR against TAA-induced liver injury by utilizing a zebrafish model and an in vitro HepG2 cell model. We also revealed that ARR attenuates oxidative stress and significantly inhibits the inflammatory response induced by the aberrant activation of the IKKβ/NF-κB signaling pathway, resulting in reduced liver injury. Furthermore, the findings will lay a foundation for the further development of *L. tanakae* and its extracts. In the future, we will continue to conduct in-depth research on the hepatoprotective mechanism of ARR.

## Figures and Tables

**Table 1:** Sequences of primer pairs used in quantitative real-time PCR.

Gene	Forward Primer (5′-3′)	Reverse Primer (5′-3′)
*β-actin*	GTGATGGACTCTGGTGATGGTGTG	AGCCACGCTCGGTCAGGATC
*rela*	CGGCAGGTGGCGATAGTGTTC	GGTCTGAGGGCAGGTACTGGAAG
*ikbaB*	GAGCTTTACCGAGGCACCACTG	AATCCAACCCGCTGTCCAAACG
*ikbkb*	GAGACCAGCATTCAGATCGCCATC	GGAACATCTCTCGCCGCAACC
*cxcl8*	CAGAAAGCCGACGCATTGGAAAAC	GAGCAGAGGGGTCCAGACAGATC

## Data Availability

Data will be made available on request.

## Supplementary Materials

The following supporting information can be downloaded at https://www.mdpi.com/article/10.3390/ph18010116/s1, Figure S1: Effect of ARR on the survival of zebrafish larvae; Figure S2: The HPLC chromatogram of rhamnetin 3-*O*-α-rhamnoside (purity ≥ 95%); Figure S3: Analysis of results related to qRT-PCR for each group; Table S1: SYBR Green I PCR systems; Table S2: SYBR Green I PCR procedures; Table S3: The concentration of RNA.
