# Supplementary material for: Investigating the Effect and Potential Mechanism of Rhamnetin 3-O-α-Rhamnoside on Acute Liver Injury In Vivo and In Vitro
Source: Pharmaceuticals (Basel). 2025 Jan 17;18(1):116. doi: 10.3390/ph18010116 (PMC11769157; doi:10.3390/ph18010116)
Supplement: Supplementary file 1 [file pharmaceuticals-18-00116-s001.zip › pharmaceuticals-3362854-supplementary.pdf]

Supplementary Material

Investigating the Effect and Potential Mechanism of Rhamnetin 3-*O*-*α*-Rhamnoside on Acute Liver Injury In Vivo and In Vitro

Dandan Deng [1], Borong Zhao [1], Hong Yang [1], Songsong Wang [2], Ziying Geng [1], Jiangtao Zhou [1], Guane Yang [1,*] and Liwen Han [2,*]

1 School of Pharmaceutical Sciences, Shanxi Medical University, No. 56 South Xinjian Road, Taiyuan 030001, China

2 School of Pharmaceutical Sciences & Institute of Materia Medica, Shandong First Medical University & Shandong Academy of Medical Science, No. 6699 Qingdao Road, Jinan 250117, China

* Correspondence:

Guane Yang (School of Pharmaceutical Sciences, Shanxi Medical University, No.56 South Xinjian Road, Taiyuan 030001, China. Tel: +86 13485383410. E-mail: yangguane@sxmu.edu.cn)

Liwen Han (School of Pharmaceutical Sciences & Institute of Materia Medica, Shandong First Medical University & Shandong Academy of Medical Science, No.6699 Qingdao Road, Jinan 250117, China. Tel: +86 156 5056 4902. E-mail: hanliwen@sdfmu.edu.cn)

Supplementary Figures and Tables

List of Figures

Supplementary Figure S1 Effect of ARR on the survival of zebrafish larvae.

Supplementary Figure S2 The HPLC chromatogram of rhamnoside 3-*O*-α-rhamnoside (purity ≥ 95%).

Supplementary Figure S3 Analysis of results related to qRT-PCR for each group.

List of Tables

Supplementary Table S1 SYBR Green I PCR systems

Supplementary Table S2 SYBR Green I PCR procedures

Supplementary Table S3 The concentration of RNA

Supplementary Figures

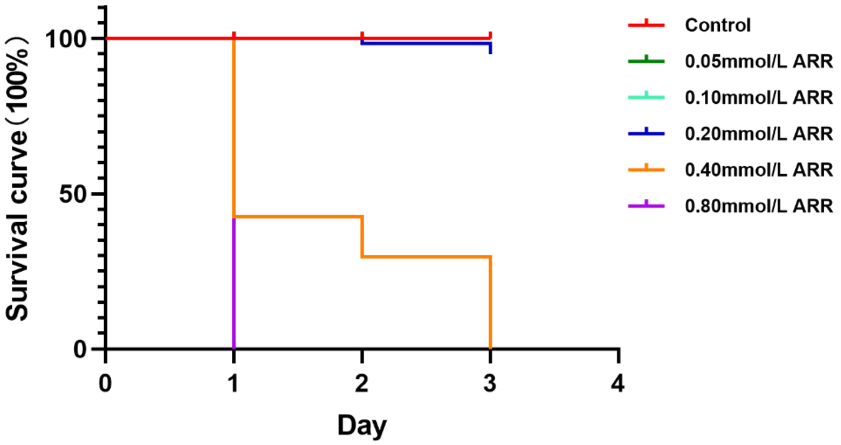

Supplementary Figure S1. Effect of ARR on the survival of zebrafish larvae.

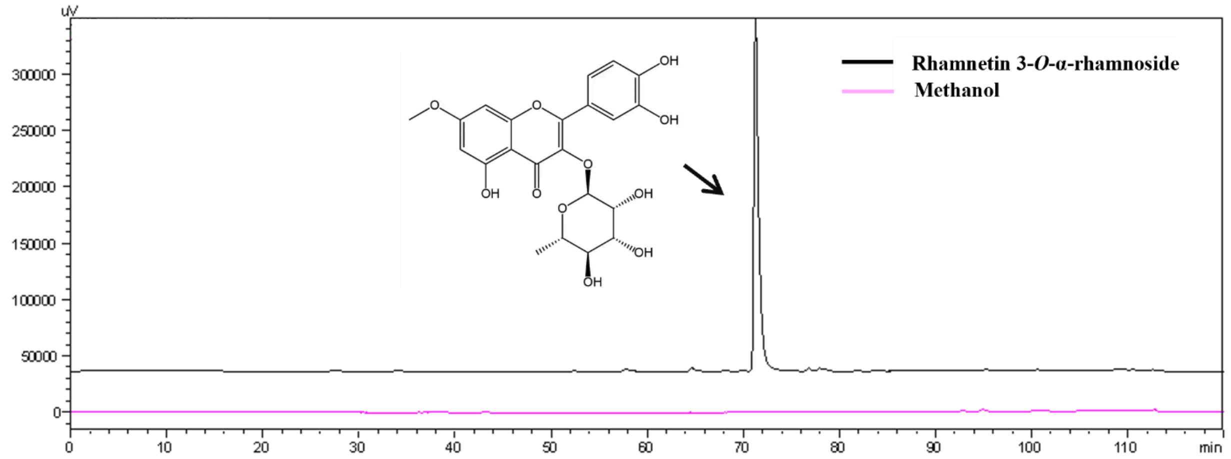

Supplementary Figure S2. The HPLC chromatogram of rhamnetin 3-*O*-α-rhamnoside (ARR) (purity ≥ 95%). HPLC separation was performed on a Shimadzu LC-30AD pump with SPD-M20A detector (Shimadzu, Kyoto, Japan) in 280 nm. For the HPLC columns, Diamonsil 5μm C18 (Dikma, China; 250 mm × 4.6 mm). The column temperature was set to 35℃. The sample injection volume was 20 μL. The mobile phase was consisted of 0.1% phosphoric acid water (A) and acetonitrile (B) at a flow rate of 1mL/min. The gradient elution procedure was as follows: 0-15 min, 5%-10% B; 15-25 min, 10%-15% B; 25-35 min, 15%-19% B; 35-60 min, 19%-30% B; 60-75 min, 30%-45% B; 75-85 min, 45%-80% B; 85-100 min, 80%-100% B.

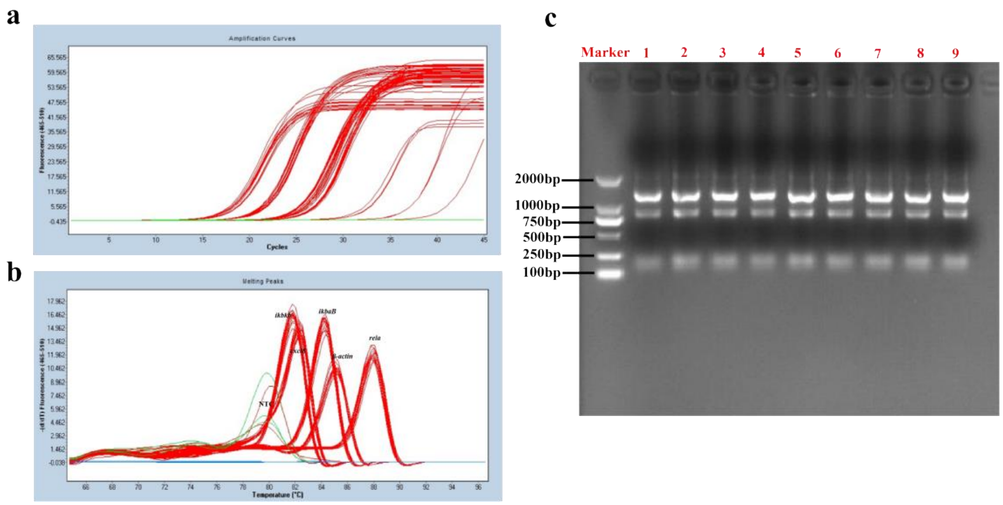

Supplementary Figure S3. Analysis of results related to qRT-PCR for each group. (**a**) Amplification curves. (**b**) Melt curves of *rela*, *ikbaB*, *ikbkb*, *cxcl8* and *β-actin*. (**c**) Results of RNA agarose gel electrophoresis. From left to right these are 2000 DNA Marker. 1: ARR1, 2: ARR2, 3: ARR3, 4: C1, 5: C2, 6: C3, 7: M1, 8: M2 and 9: M3.

Supplementary Tables

Supplementary Table S1. SYBR Green I PCR systems

Reagents	Additions
2×SYBR Green qPCR Mix	10 μL
Primer F (10 μM)	0.4 μL
Primer R (10 μM)	0.4 μL
cDNA	1 μL
RNase Free H_2O	up to 20 μL

Supplementary Table S2. SYBR Green I PCR procedures

Procedures	Temperature	Time
**Pre-incubation**	95 °C	3 min
**Amplification (45 cycles)**	95 °C	20 s
	61 °C	15 s
**Melting curves**	95 °C	5 s
	65 °C	1 min
	97 °C	Continuous

Supplementary Table S3. The concentration of RNA

Sample Name	OD260/280	OD260/230	Concentration (ng/μL)	Tape
**ARR 1**	1.96	1.77	1000.94	RNA
**ARR 2**	1.94	1.79	1346.89	RNA
**ARR 3**	1.92	1.68	1244.69	RNA
**CON 1**	1.88	1.66	1220.49	RNA
**CON 2**	1.92	1.56	1384.25	RNA
**CON 3**	1.95	1.74	839.509	RNA
**MOD 1**	1.9	1.6	909.244	RNA
**MOD 2**	1.83	1.35	851.17	RNA
**MOD 3**	1.9	1.5	826.627	RNA
